# From shape to contents: heterogeneity of CNS glial cells

**DOI:** 10.1007/s00401-021-02398-w

**Published:** 2021-12-15

**Authors:** Chintan Chhatbar, Marco Prinz

**Affiliations:** 1 Institute of Neuropathology, Faculty of Medicine, University of Freiburg, Breisacher Str. 64, 79106 Freiburg, Germany; 2 Signalling Research Centres BIOSS and CIBSS, University of Freiburg, Freiburg, Germany; 3 Center for Basics in NeuroModulation (NeuroModulBasics), Faculty of Medicine, University of Freiburg, Freiburg, Germany

Central nervous system (CNS) comprises of heterogeneous cell types which include neurons and glia. Glia stands for the Greek word “glue” indicating that these cells were initially considered purely as non-functional bystander cells proving neurons only their structural context. This simplified view changed dramatically in the last decades and glial cells are nowadays considered as highly functional cells during development, homeostasis and disease. Among the glia, microglia are yolk sac derived, whereas astrocytes and oligodendroglia are neuroectoderm derived [3, 4]. Although recent advances in multiomic technologies have expanded about how these cells behave in a healthy CNS and react to diseases, there is still a remarkable gap in the knowledge about region-specific temporal heterogeneity of glia. As for the factors that generate the heterogeneity, the microenvironment could be one major factor which could potentially give rise to differences, e.g., between the white and gray matter glia. However, it still remains to be addressed if there is heterogeneity across diverse white/gray matter regions of the CNS and how these differences could further promote CNS functioning and diseases.

In the context of the current review cluster in this issue of Acta Neurpathologica, several of the above-mentioned key points are addressed. Amor et al*.* discuss the current state of the art with respect to microglia development, heterogeneity of states and functions, and specific role(s) of white matter microglia in CNS development and the diseases where white matter is affected [1]. Seeker et al*.* dive into the heterogeneity of human oligodendroglia and the future challenges on how to push the field forward [6]. Bugiani et al*.* describe the white matter astrocyte heterogeneity and discuss their role in towards development of astrogliopathies [2]. Schwabenland et al*.* discuss current state of microglia heterogeneity and provide a practical guide for neuroscientist in general on how to analyze microglial phenotypes in depth in common neuropathologies [5]. Finally, Spiteri et al*.* put microglia vis-à-vis monocytes in several neuropathologies to discuss their partially redundant and non-redundant functions which are temporally regulated [7]. In sum, it is tempting to speculate that the identification of context-dependent glial states and/or subpopulations might pave the way for novel therapeutic strategies in the future [3].
